# Vitrectomy without prone positioning for rhegmatogenous retinal detachments in eyes with inferior retinal breaks

**DOI:** 10.1371/journal.pone.0191531

**Published:** 2018-01-26

**Authors:** Nobuhiko Shiraki, Susumu Sakimoto, Hirokazu Sakaguchi, Kentaro Nishida, Kohji Nishida, Motohiro Kamei

**Affiliations:** 1 Department of Ophthalmology, Osaka University Graduate School of Medicine, Department of Ophthalmology, Osaka, Japan; 2 Department of Ophthalmology, Aichi Medical University, Nagakute, Japan; Massachusetts Eye & Ear Infirmary, Harvard Medical School, UNITED STATES

## Abstract

**Purpose:**

To compare the anatomic and functional outcomes of pars plana vitrectomy (PPV) for treating rhegmatogenous retinal detachments (RRDs) between two groups with and without postoperative prone positioning.

**Methods:**

This retrospective cohort study included 142 eyes of 142 patients with a primary RRD. All patients underwent PPV with 20% sulfur hexafluoride gas tamponade and were divided into two groups: the groups that did and did not maintain a prone position postoperatively. All patients were followed for more than 3 months. The main outcome measures were the best-corrected visual acuity (BCVA), retinal reattachment rate, and postoperative complications.

**Results:**

Sixty-five eyes were included in the prone position group and 77 eyes in the group without prone positioning; the respective initial reattachment rates were 83.1% and 96.1%, a difference that reach significance (p = 0.011). In the eyes with inferior breaks, the initial reattachment rate was 94.7% (18 eyes) without prone positioning, which was significantly (p = 0.036) better than the 60% (6 eyes) initial reattachment rate in the group with prone positioning. In the eyes without inferior breaks, there was no significant difference in the initial reattachment rates between the two groups. The BCVAs at the 3-month postoperative visit did not differ significantly between the two groups. An epiretinal membrane (ERM) was observed postoperatively in 10 (13.0%) eyes in the group without prone positioning; no ERMs were seen postoperatively in eyes in which the internal limiting membrane (ILM) was peeled during PPV.

**Conclusions:**

PPV without postoperative prone positioning is associated with a higher reattachment rate in eyes with a RRD, especially those with inferior retinal breaks. PPV with postoperative supine and lateral positioning might be beneficial to manage RRDs associated with inferior retinal breaks if ILM peeling is performed intraoperatively.

## Introduction

Pars plana vitrectomy (PPV) with gas tamponade is the surgery performed most frequently to treat rhegmatogenous retinal detachments (RRDs) in developed countries. Recent advances in technologies used during PPV, i.e., smaller gauge instrumentation, wide-angle viewing systems, and high-speed vitreous cutters, have enabled surgeons to perform fewer invasive surgeries with shorter operating times to minimize surgical invasiveness and patient discomfort. However, gas tamponade still involves uncomfortable prone positioning postoperatively.

PPV and gas tamponade without face-down positioning have been performed to treat macular holes (MHs) [1,2]; nonetheless, few studies have reported the outcomes after PPV to treat RRDs without postoperative prone positioning. Martínez-Castillo et al. reported the results of a consecutive non-comparative study of pseudophakic eyes [3] and Chen et al. performed a comparative study of PPV using long-acting gas [4]. In the current study, we retrospectively compared the results achieved with prone positioning with the results without prone positioning after primary PPV to treat RRDs in a larger case series.

PPV combined with cataract surgery, known as phacovitrectomy, is performed widely in old patients with vitreoretinal diseases [5–8]. The advantages of combined phacovitrectomy include faster visual recovery compared with that after two separate procedures, safe vitreous shaving without concern for intraoperative lenticular touch or postoperative cataract progression [9–12], and reduced surgical time and cost [13]. In the current study, we also included eyes treated with combined phacovitrectomy, which is being performed frequently [14], to establish a less invasive surgical approach in eyes with RRDs.

## Patients and methods

We analyzed retrospectively the medical records of 142 eyes of 142 consecutive patients who underwent PPV to treat RRDs. The Osaka University Hospital approved this study, which was conducted from March 2013 to June 2015. All data were fully anonymized before we accessed them and the IRB waived the requirement for informed consent. Four surgeons (MK, HS, KN, and SS) performed all surgical procedures. The exclusion criteria were a history of surgery for any retinal diseases, proliferative vitreoretinopathy of grade C or worse, giant retinal tears, or myopic MHs.

PPV was performed using a 25-gauge system (Constellation and Accurus, Alcon Laboratories Inc., Fort Worth, TX, USA). A three-port PPV was performed using a wide-angle viewing system (Resight, Carl Zeiss Meditec, Oberkochen, Germany). Simultaneous cataract surgery (phacoemulsification and intraocular lens [IOL] implantation) was performed in all phakic eyes. After core vitrectomy, triamcinolone acetonide (MaQaid, Wakamoto Pharmaceutical, Tokyo, Japan) was sprayed toward the optic disc and the posterior retinal surface to ascertain the presence of a posterior vitreous detachment. The peripheral vitreous was shaved as much as possible under scleral indentation. In some eyes, liquid perfluorocarbon (Perfluoron, Alcon Laboratories Inc.) was used to stabilize the detached retina. Internal limiting membrane (ILM) peeling also was performed in some cases. After vitreous shaving, fluid-air exchange and endophotocoagulation were performed around all retinal tears and lattice degeneration. After the retina was reattached completely, an air-gas (20% sulfur hexafluoride [SF_6_]) exchange was performed.

The patients in this case series were divided into two groups based on those who were instructed to maintain prone positioning postoperatively and those who were not. The patients in the latter group were instructed to avoid maintaining the original retinal breaks in the lowest position and to remain in a supine position while asleep. For example, patients with an inferior break were instructed to maintain in a supine and lateral position; however, patients with a superior break were not instructed to maintain a specific posture during the daytime. Ocular examinations were performed daily until 1 week and at 1 and 3 months postoperatively. The visual acuity (VA), anatomic reattachment, and complications were assessed at 1 week and 1 and 3 months postoperatively.

Statistical analysis was performed using JUMP version 11.2.0 (SAS System, Cary, NC, USA). Continuous values were expressed as the mean ± standard deviation. The VAs were converted to the logarithm of the minimal angle of resolution (logMAR) values for all calculations. Data were analyzed using Fisher’s exact test or the Pearson chi-square test for categorical variables, and the unpaired *t*-test and the Mann-Whitney *U*-test for numerical variables. P values less than 0.05 were considered statistically significant.

## Results

One hundred and forty-two eyes of 142 patients (mean age, 60.0 years; range, 33–87) with RRDs were followed for a mean of 8.5 months (range, 3–24). Ninety-one (64.1%) patients were men, and 51 (35.9%) were women. Forty eyes were pseudophakic and 102 eyes were phakic. The mean number of quadrants affected was 2.1 (range, 1–4). In 66 (46.5%) eyes, the macula was detached preoperatively. Twenty-nine (20.4%) eyes had breaks in the inferior quadrant, and 49 (34.5%) eyes had multiple breaks. The prone position group included 65 eyes and the non-prone position group included 77 eyes. There was no significant difference in the preoperative characteristics between the two groups (Table 1).

**Table 1:** Baseline patient characteristics with prone positioning versus no prone positioning.

	Prone Positioning (n = 65)	No Prone Positioning (n = 77)	P Value
Age (mean ± SD years)	59.7±11.8	60.3±11.4	0.980
Gender (male/female)	44 (67.7)/21 (32.3)	47 (61.0)/30 (39.0)	0.410
Preoperative BCVA (mean ± SD, logMAR)	0.59±0.72	0.50±0.78	0.200
Macular status			0.550
On	33 (50.8)	43 (55.8)	
Off	32 (49.2)	34 (44.2)	
Lens status			0.530
Phakic	45 (69.2)	57 (74.0)	
Pseudophakic	20 (30.8)	20 (26.0)	
Location of breaks			0.170
Inferior	10 (18.2)	19 (24.7)	
Not inferior	55 (81.2)	58 (75.3)	
Area of RD (mean ± SD, quadrant)	2.23±0.86	1.98±0.91	0.060

The data are expressed as numbers (%) unless otherwise indicated.

Overall, initial reattachment occurred in 128 (90.1%) eyes; the final reattachment rate was 100%. The mean final postoperative BCVA improved significantly (p<0.0001) from the preoperative BCVA. Initial reattachment in the non-prone position group was achieved in 74 (96.1%) eyes, which was significantly (p = 0.011) better than in the prone position group, in which 54 (83.1%) eyes achieved reattachment (Table 2). There was no significant (p = 0.089) difference between the two groups in the initial reattachment rate in eyes without inferior breaks. However, in eyes with inferior breaks, a significantly (p = 0.036) higher initial reattachment rate was achieved in the non-prone position group, i.e., 18 (94.7%) eyes compared with six (60%) eyes in the prone position group (Table 2). Table 3 shows the subgroup analysis of the preoperative characteristics of eyes with inferior breaks based on prone positioning or no prone positioning postoperatively. Only the area of the RD was significant. However, there was no significant difference in the extent of the RD between eyes that achieved successful reattachment and those that failed to achieve reattachment (Table 2).

**Table 2:** Risk factors for recurrence of RD in the subgroup of inferior breaks.

	Total	Prone Position	No Prone Position	p Value
Macular status				
On	15 (51.8)	3 (60.0)	12 (50.0)	
Off	14 (48.2)	2 (40.0)	12 (50.0)	1.000
Lens status				
Phakic	21 (72.4)	4 (80.0)	17 (70.8)	
Psudophakic	8 (27.6)	1 (20.0)	7 (29.2)	1.000
Number of break				
Single	11 (37.9)	2 (40.0)	9 (37.5)	
Multiple	18 (62.1)	3 (60.0)	15 (62.5)	1.000
Area of RD				
(mean±SD, quadrant)	2.2 ± 0.9	2.6 ± 1.1	2.13 ± 0.8	0.270

RD, retinal detachment

The data are expressed as the number (%) unless otherwise indicated.

**Table 3:** Postoperative outcome measures after vitrectomy with prone positioning versus without prone positioning.

	Prone Position	No Prone Position	p Value
Initial reattachment rate (%)	54/65 (83.1)	74/77 (96.1)	0.011
Eyes without inferior breaks	48/55 (87.3)	56/58 (96.6)	0.089
Eyes with inferior breaks	6/10 (60)	18/19 (94.7)	0.036
Final reattachment rate (%)	65 (100)	77 (100)	1.000
BCVA (logMAR) 3 months after vitrectomy (mean ± SD)	0.14±0.31	0.16±0.41	0.580
IOP elevation (>22 mmHg)	15 (23.1)	13 (16.9)	0.360
ERM on macula after vitrectomy	2 (3.1)	10 (13)	0.039
Combined ILM peeling during vitrectomy	5/65 (7.7)	14/77 (18.2)	0.085
ERM occurrence in eyes with ILM peeling	0/5	0/14	
ERM occurrence in eyes without ILM peeling	2/60 (3.3)	10/63 (15.6)	0.030
Fibrin formation in anterior chamber on postoperative day 1	17 (26.2)	10 (13)	0.046
IOL optic capture after vitrectomy	3 (4.6)	2 (2.6)	0.520
MH after vitrectomy	0 (0)	1 (1.3)	1.000
PVR after vitrectomy	1 (1.5)	1 (1.3)	1.000

The data are expressed as the number (%) unless otherwise indicated.

Intraocular pressure (IOP) elevations over 22 mmHg occurred in 28 (19.7%) of 142 eyes. IOL capture was detected in five (3.5%) eyes. A MH developed in one (1.3%) eye in the non-prone position group. Proliferative vitreoretinopathy (PVR) was observed in one (1.3%) eye in the non-prone position group and one (1.5%) eye in the prone position group. There was no significant difference in the incidence of IOP elevations, IOL capture, MHs, and PVR between the two groups (p = 0.36, p = 0.52, p = 1.000, and p = 1.000, respectively) (Table 3). Fibrin was present in the anterior chamber the first day after vitrectomy in 17 (26.2%) eyes in the prone position group, which was significantly (p = 0.046) higher than in 10 (13.0%) eyes in the non-prone position group (Table 3). The rate of development of postoperative ERMs on the macula was relatively (p = 0.039) higher in the non-prone position group (10 eyes, 13%) compared to the prone position group (2 eyes, 3.1%) (Table 3). However, postoperative ERMs were not observed in eyes in both groups in which ILM peeling also was performed.

In all phakic eyes that underwent phacovitrectomy, initial reattachment in the non-prone position group was achieved in 55 (96.5%) eyes, which was significantly (p = 0.021) better than in the 37 (82.2%) eyes in the prone position group (Table 4). There was no significant (p = 0.412) difference between the two groups in the initial reattachment rates in eyes without inferior breaks; in eyes with inferior breaks, the initial reattachment rate in the non-prone position group was significantly (p = 0.006) higher in the 14 (100%) eyes compared with the three (42.9%) eyes in the prone position group. There also was no significant (p = 1.000) difference in the rate of IOL capture (Table 5). Data set which was used on this study is available (S1 File).

**Table 4:** Subgroup analysis of baseline characteristics in patients with inferior breaks.

	Prone Positioning (n = 10)	No Prone Positioning (n = 19)	p Value
Macular status			0.450
On	4 (40.0)	11 (57.9)	
Off	6 (60.0)	8 (42.1)	
Lens status			1.000
Phakic	7 (70.0)	14 (73.7)	
Pseudophakic	3 (30.0)	5 (26.3)	
Number of break			0.694
Single	3 (30.0)	8 (42.1)	
Multiple	7 (70.0)	11 (57.9)	
Area of RD (mean±SD, quadrant)	2.9 ± 0.9	1.8 ± 0.6	0.003

SD = standard deviation.

The data are expressed as numbers (%) unless otherwise indicated.

**Table 5:** Outcome measures after prone positioning following vitrectomy compared with no prone positioning in eyes that underwent phacovitrectomy.

	Prone Positioning	No Prone Positioning	p Value
Initial reattachment rate (%)	37/45 (82.2)	55/57 (96.5)	0.021
Eyes without inferior breaks	34/38 (89.5)	41/43 (95.3)	0.412
Eyes with inferior breaks	3/7 (42.9)	14/14 (100)	0.006
Final reattachment rate (%)	55 (100)	58 (100)	1.000
BCVA (logMAR) 3months after vitrectomy, (mean ± SD)	0.12±0.3	0.17±0.39	0.833
IOP elevation (>22 mmHg)	13 (28.9)	10 (17.5)	0.233
ERM on macula after vitrectomy	2 (4.4)	8 (14)	0.180
Combined ILM peeling during vitrectomy	4/45 (8.9)	12/57 (21.1)	0.108
ERM occurrence in eyes with ILM peeling	0/4	0/12	
ERM occurrence in eyes without ILM peeling	2/41 (4.9)	8/45 (17.8)	0.092
Fibrin formation in anterior chamber on postoperative day 1	14 (31.1)	8 (14)	0.037
IOL optic capture after vitrectomy	1 (2.2)	2 (3.5)	1.000
MH after vitrectomy	0 (0)	1 (1.7)	1.000
PVR after vitrectomy	1 (2.2)	1 (1.8)	1.000

SD = standard deviation.

The data are expressed as the number (%) unless otherwise indicated.

## Discussion

In the current study, we compared the surgical outcomes after PPV in eyes with and without postoperative prone positioning. The overall primary retinal attachment rate in this study was 90.2%, which compared favorably with those reported previously [15–17]. The initial reattachment rate in the non-prone position group was significantly higher than in the prone position group. Particularly in eyes with inferior breaks, the non-prone position group had a much higher retinal reattachment rate than in the prone position group, while in eyes without inferior breaks, there was no significant difference in the initial reattachment rate between the two groups.

The belief is that closure of retinal breaks depends on the effect of surface tension rather than buoyancy by gas tamponade [18]. Indeed, multiple groups have reported the efficacy of postoperative positioning without maintenance of a facedown position after vitrectomy for MHs [1,2]. However, only a few groups have reported the efficacy of not maintaining a facedown position after vitrectomy to manage RDs [19]. The principle of those studies was based on the effect of gas tamponade, i.e., surface tension rather than buoyancy, which prevents access of the intraocular fluid to the subretinal space through the retinal breaks [20]. Some reports have argued the relevance of intraoperative subretinal fluid drainage, in which simple gas coverage of the retinal breaks after sufficient photocoagulation is a key feature in creating chorioretinal adhesion [21]. The current study showed the superiority of the anatomic success rate in eyes with inferior breaks in the group that maintained a supine or lateral position compared with the group that maintained prone positioning. Practically speaking, intraocular gas might not come into contact with the original break during incomplete prone positioning in eyes with inferior tears. In addition, Bell’s phenomenon might result in the exposure of breaks to intravitreal fluid and not to gas during sleep while the patient is in the facedown position. Therefore, our hypothesis that includes supine positioning recognizes a novel postoperative strategy for managing inferior RDs.

To enhance the success rates of PPV for RRDs with inferior retinal breaks, several treatment options, i.e., PPV with an encircling band [22], use of long-acting tamponades [23], silicone oil [24], and strict positioning of the patient during the postoperative period, have been reported [23]. Even though Duvdevan et al. reported that no significant difference was found between break locations when superior and inferior breaks were compared [25], the presence of an inferior tear still is regarded as a risk factor for postoperative recurrence of RRDs. The combination of scleral buckling and/or silicone oil tamponade during vitrectomy has been reported to be effective in eyes with inferior tears [15,26]; however, that combination has been inconsistent when considering the trend toward non-invasive vitrectomy [15,25]. Although use of a long-acting gas such as 14% octafluoropropane (C_3_F_8_) or a dense gas such as 25% SF_6_ during vitrectomy for eyes with RRDs also has been reported, Wong et al. reported that the IOP increased over 30 mmHg in over 20% of patients and that about 5% of patients had IOPs that increased over 40 mmHg when 16% C_3_F_8_ or 30% SF_6_ was used during vitrectomy[20].

Regarding complications after vitrectomy, the current rate of development of ERMs on the macula, 8.4% (12 eyes), was comparable to those reported previously (6%-12.8%) [27–29]. The rate of development of ERMs in the group without prone positioning was higher than in the group with prone positioning, i.e., 12.8% (10 eyes) compared with 3.1% (2 eyes), respectively.

Fibrin developed significantly more often in the anterior chamber 1 day after vitrectomy in the group with prone positioning (26.2%) compared with the non-prone position group (12.8%). In eyes filled with gas, inflammatory cells and cytokines should accumulate inferiorly after vitrectomy, which presumably might accelerate ERM formation in the eyes of patients who maintained a supine position, and fibrin in the eyes of patients who maintained prone positioning. In the current study, we detected more ERMs on the maculas in the non-prone position group, which presumably was due to supine positioning. However, Table 2 shows that ILM peeling prevented postoperative ERM formation.

The current study was the first to compare the effects of postoperative positioning in eyes that underwent combined phacovitrectomy to treat RRDs. The initial reattachment rate in the non-prone position group was significantly higher than in the prone position group. Only the eyes with inferior breaks in the non-prone position group had a better retinal reattachment rate compared with prone positioning. No factor except the area of the RD differed between the two groups in the subset of inferior breaks (Table 3). However, the eyes in which reattachment was not achieved did not have a larger area of RD (S1 File). Even though the eyes in the group that maintained a supine position and not a facedown position underwent cataract surgery, there was no significant difference in the rate of postoperative IOL optic capture between the two positions.

The major limitations of the current study were its relatively small sample size and the retrospective, non-randomized design. Even though there were no significant differences in the preoperative parameters, the areas of the RDs were larger in the prone position group, which might have affected the anatomic success rate.

In conclusion, this retrospective study suggested that maintaining a strict prone position, which potentially induces physical and mental burdens on the patient, is not required in pseudophakic eyes or after phacovitrectomy. Positioning without use of a strict prone position did not decrease the success rate of vitrectomy performed to treat RRDs, rather, a supine or lateral position seemed to be effective for eyes with inferior RDs. Randomized and prospective studies with larger sample sizes are warranted to further determine the efficacy of postoperative positioning without a strict prone position for managing RRDs.

## Supporting information

S1 File Demographic data and outcomes of vitrectomy in this series. S1 file contains data from 147 cases of RRD included in this study.
(XLSX)
